# Income loss and gender-based violence during the COVID-19 pandemic among female entertainment workers in Cambodia: a cross-sectional phone survey

**DOI:** 10.1186/s12889-023-15044-9

**Published:** 2023-02-08

**Authors:** Carinne Brody, Natasha Harrison, Siyan Yi

**Affiliations:** 1 College of Education and Health Sciences, Public Health Program, Touro University California, Vallejo, CA USA; 2 School of Public Health, University of California, Berkeley, CA USA; 3 Saw Swee Hock School of Public Health, National University of Singapore and National University Health System, Singapore, Singapore

**Keywords:** COVID-19, Economic impact, Entertainment work, Gender-based violence, Food security, Mental health

## Abstract

**Background:**

In Cambodia, female entertainment workers (FEWs) are disproportionately affected by global and local disasters, such as the COVID-19 pandemic. To prevent the spread of COVID-19, the government imposed tight restrictions, including closures of entertainment venues, such as karaoke bars, beer gardens, nightclubs, or massage parlors, leading FEWs to face economic and social disruptions. This study aims to assess the relationship between income loss during the pandemic and gender-based violence (GBV) among FEWs in Cambodia to inform future disaster response programs.

**Methods:**

We conducted a phone survey in August 2021 with 369 randomly sampled FEWs from a national organization’s email list. We used a structured questionnaire to ask the participants about job and income loss, food security, mental health, access to health services, and GBV. We fit a linear regression model to examine the differences in GBV experience between FEWs who lost all their income and those who lost partial income due to the COVID-19 pandemic. Key covariables comprised the number of dependents, smartphone ownership, internet access, food security, and mental health. Multivariable linear regression analysis was conducted.

**Results:**

The mean age (31.6 vs. 30.6), years of formal education (6.3 vs. 6.3), marital status (24.2 vs. 23.8 never married), and the number of children (1.3 vs. 1.1) of women reporting having lost all income were not significantly different from those who experienced partial income loss. Overall, GBV experiences were significantly higher in FEWs who lost all income than in those who lost partial income (62.9% vs. 47.4%, *p* = 0.03). Controlling for the number of dependents, smartphone ownership, and food security, the adjusted odds ratio for GBV was significant in the adjusted model (AOR = 1.23 (1.08–1.40), *p* = 0.001) indicating that those who experienced total income loss were more likely to experience GBV than those who experienced partial income loss. In addition, they were significantly less likely to be food secure (*p* = 0.04), less likely to own a smartphone (*p* = 0.02), and had more dependents (*p* < 0.001).

**Conclusion:**

Disaster response programs should consider the implications of safety measures and government support for both formal and informal workers regarding safety, food access, and mental health support. Food assistance programs should target the most vulnerable informal sector workers during crises.

## Background

Global crises, such as natural disasters, disease outbreaks, and economic downturns, adversely affect the entire population. However, marginalized groups are disproportionately impacted by such situations [1]. Public health measures intended to maintain and ensure safety during a crisis can inadvertently exacerbate the underlying social and health inequities marginalized groups experience by increasing barriers to accessing health and social services [2]. One crucially marginalized and understudied group is female entertainment and sex workers [3].

In Cambodia, female entertainment workers (FEWs) include young women employed at establishments, such as karaoke bars, beer gardens, nightclubs, or massage parlors, who may engage in transactional sex with clients to support their livelihoods [4]. Sex work was made illegal in Cambodia in 2008, and entertainment workers have experienced stigmatization and discrimination [5]. To prevent the spread of COVID-19 during the 2020 pandemic, the Cambodian government, like many other governments across the world, imposed tight restrictions on work and social life. Entertainment venues were, therefore, shut down across the county [6–8]. As a result, FEWs faced economic and social disruptions like other marginalized populations [6–9]. When entertainment venues were shut down, services aimed to support FEWs were temporarily closed or limited [9].

In other countries that experienced pandemic shutdowns, FEWs and sex workers struggled with economic hardship and experienced more barriers to accessing essential healthcare [10]. Despite these adversities, some FEWs in Cambodia continued to visit with clients against lockdown rules [11]. During these visits, FEWs reported feeling at risk for gender-based violence (GBV) due to the lack of security and support regarding safe sex negotiations [10–12]. Furthermore, FEWs were not always eligible for government assistance due to the informal nature of their jobs [13, 14]. Because the profession of sex work through entertainment venues remains illegal in Cambodia, sex workers cannot access protection from law enforcement if a crime has been committed against them [13]. During government closures, FEWs that operate outside of their usual entertainment venues without the protection afforded by a formal workplace establishment are more vulnerable to workplace GBV [14]. In addition, job loss and lack of income are known to increase the potential for domestic violence [15, 16].

In order to understand the consequences of the pandemic shutdown on a vulnerable group, this study aims to assess the impact of income loss due to the COVID-19 pandemic on GBV among FEWs in Cambodia.

## Methods

This cross-sectional phone survey was conducted in August and September 2021 as a need assessment and situation analysis to inform the implementation of a community-based GBV intervention among FEWs. The study used a structured questionnaire administered by outreach workers trained as data collectors. We had access to a master list of 3000 FEWs kept by a local community-based organization and aimed to collect a minimum sample of 341. This was based on a sample size calculation using 5% margin of error, 95% confidence interval (CI) and a 50% response distribution since we did not know how many FEWs had lost total income during the pandemic. In order to decrease our margin of error, we aimed for 370 participants. Using a random number generator, we randomly selected 370 FEWs. If we could not reach a selected FEW, we would generate another random number until we reached our target number of 370. We invited them to participate through phone recruitment using a standardized script. We included women aged 18 and older who worked at entertainment venues in the capital city of Phnom Penh. We excluded ​minors younger than 18, those working outside entertainment venues before the lockdown, such as on the street, and those unable to provide verbal informed consent. Respondents received USD5 after the interview for their time and effort compensation.

### Data collection procedures

Data collectors signed the consent form to confirm that the study participants had been briefed about the study, assured of their confidentiality, and consented to participate in the interview. The data collectors entered the data via an online survey platform called Kobo Collect Toolbox. During the phone interview, participants were asked close-ended questions from a questionnaire with a few open-ended questions to clarify their situation. Each interview lasted 30–45 min.

### Variables and measurements

The questionnaire included questions about participants’ socioeconomic characteristics and life stressors exacerbated by the COVID-19 pandemic, including COVID-19 perceptions, employment changes, loss of income, food security, housing security, GBV, healthcare access, and mental health.

*Socioeconomic characteristics* included age, education, marital status, number of children, smartphone ownership, daily internet access, and personal finance questions, including income before and after 2020, number of dependents, work at entertainment venues before 2020, need to acquire a new job after 2020, and borrowed money from someone since 2020.

*Gender-based violence* GBV experience was measured using a 6-item questionnaire measured on a Likert scale based on the World Health Organization’s GBV prevalence surveys, which have been used globally [17]. Each GBV-related question was measured with three-point response categories from “no” to “yes, once” to “yes, multiple times” concerning the frequency of GBV experience. The 6 items were averaged into a composite score ranging from 1 to 3. Higher values on this scale represent higher levels of GBV experience. The following are the 6 items in the questionnaire:

1. Has a partner tried to restrict (online or phone) contact with your family?
2. Has a partner insulted you or made you feel bad about yourself?
3. Has a partner ever not provided you money to run the house or look after the children but provided money for other things?
4. Has a partner slapped, pushed, hit, kicked, or choked you or thrown something at you that could hurt you?
5. Has a partner physically forced you to have sex when you did not want to?
6. Has a partner made you have sex when you did not want to because you were afraid of what your partner might do?

*Food security* was measured in a 4-item questionnaire that assessed food security and hunger experience adapted from the United States Department of Agriculture (USDA) Six-Item Short Form of the Food Security Survey Module, which has been validated in developing country contexts [18]. Respondents were asked about the frequency of feeling worried about their food running out before obtaining money to buy more, how often they forwent eating balanced meals due to financial restraints, how often they cut the size of or skipped their meals, and how often they went an entire day without eating in the last 12 months. The food security index was measured with five-point response categories from ‘always’ to ‘never.’ Composite scores were calculated for food security and ranged from 1 to 5, with a lower score indicating greater food insecurity experience. The 4 items in this section were:

1. How often did you worry whether your food would run out before you got money to buy more in the last 12 months?
2. How often did you not eat balanced meals because you could not afford it in the last 12 months?
3. How often did you cut the size of your meals or skip meals because there was not enough money for food in the last 12 months?
4. How often did you not eat for a whole day because there was not enough money for food in the last 12 months?

*Mental health* Lastly, we measured mental ill-health with a validated General Health Questionnaire (GHQ-12) that has been validated in Asian populations [19]. This 12-item self-report questionnaire was designed to assess the severity of psychological distress. The items were measured with a four-point response scale. A mental health composite score was then computed from the 12-items with higher scores representing increased depressive symptoms. Mental health composite scores ranged from 1 to 4, with a higher score indicating greater mental ill-health. This item was used as a continuous variable.

### Statistical analyses

Data were entered in Microsoft Excel for cleaning and imported into STATA16.1 for analyses.

Summary statistics for categorical socioeconomic variables were reported as frequencies and percentages, while means and standard deviations (SD) were used as summary statistics for continuous variables. Bivariate Chi-square analyses and *t*-tests were used to compare potential confounder variables between participants who lost their total income to those who lost only a part of their income due to the COVID-19 pandemic (Table 1). We then contracted an adjusted multivariable linear regression model to assess the association between income loss and GBV composite scores. The covariables in the model included daily internet access, whether the participant owned a smartphone or tablet, the number of dependent family members, mental health score (GHQ12), and food security score. Confounders were identified through a directed acyclic graph (DAG), a review of prior literature from similar studies, and results from the bivariate analyses. Interactive effects of food security and GBV were assessed by adding an interaction term (food security*gbv) to our final model. Internet access and smartphone ownership for women were associated with increased gender equity and improved educational and social outcomes, including GBV [20–22]. We included the number of dependents because the need to financially support dependents has been linked to engagement in sex work and low negotiating power with clients due to constrained choices which are risk factors for GBV [23–25].

**Table 1:** Socioeconomic characteristics of the study population (*n* = 369)

**Socioeconomic characteristics**	**Total income loss (*n* = 62)**	**Partial income loss (*n* = 307)**	***P*-value**
Age (mean ± SD)	31.6 ± 7.8	30.6 ± 7.4	0.34
Years of formal education (mean ± SD)	6.3 ± 3.7	6.3 ± 3.3	0.94
Marital status (*n*, %)			0.57
Never married	15 (24.2)	73 (23.8)	
Never married but living with a partner	3 (4.8)	19 (6.2)	
Married and living with a partner	25 (40.3)	93 (30.3)	
Married but not living with a partner	3 (4.8)	20 (6.5)	
Divorced/separated/widowed	16 (25.8)	102 (33.2)	
Number of children (mean ± SD)	1.3 ± 1.5	1.1 ± 1.2	0.20
Own a smartphone or tablet (*n*, %)	49 (79.0)	289 (94.1)	< 0.001*
Daily internet access (*n*, %)	48 (77.4)	275 (89.6)	0.02*
Monthly income before 2020 (mean ± SD)	274.7 ± 184.5	385.3 ± 254.3	0.001*
Monthly income since 2020 (mean ± SD)	0.0 ± 0.0	194.4 ± 117.5	< 0.001*
Number of dependent family members before 2020 (mean ± SD)	3.1 ± 1.7	3.4 ± 1.9	0.18
Number of dependent family members since 2020 (mean ± SD)	2.0 ± 2.1	3.2 ± 1.8	< 0.001*
Work at any entertainment establishments before 2020 (*n*, %)	62 (100.0)	303 (98.7)	0.82
Needed to acquire a new or second job to gain income (*n*, %)	24 (38.7)	113 (36.8)	0.89
Borrowed money from someone since 2020 (*n*, %)	38 (62.3)	181 (59.0)	0.73
Experienced any GBV (*n*, %)	39 (62.9)	145 (47.4)	0.03*
Experienced physical or sexual violence (*n*, %)	14 (22.6)	40 (13.1)	0.05*
Food security score (mean ± SD)	12.3 ± 4.0	13.7 ± 3.4	0.002*
Mental health score (mean ± SD) (*n* = 196)	24.3 ± 5.5	24.9 ± 4.4	0.50
GBV score (mean ± SD)	10.2 ± 4.1	8.4 ± 2.1	< 0.001*

*GBV* Gender-based Violence, *SD* standard deviation

*statistical significance at *p*<0.05

We then examined the interactive effects between food security and GBV in the adjusted analyses using the original model plus an interaction term (food security*GBV). We also computed the marginal effect of food security using the *dydx* option in STATA. A margins plot was generated to display the marginal effects of this interaction.

### Ethical issues

Before data collection, verbal informed consent was sought from the study participants. Because this research topic is highly stigmatizing and the average literacy level in this population was low, verbal consent was the most appropriate form to ensure that no other identifying information was collected. In addition, the ethics committee approved verbal consent due to restrictions during the pandemic lockdown. Participants were reminded about the voluntary nature of their participation and their right to end their participation at any point during the phone interview without any consequences. Participants were informed of free counseling services and information about crisis centers and other relevant referrals. The records of this study were kept confidential. The research team did not include the name or personal identifiers of any participants involved in the research in the reports. The information obtained from the participants was uploaded onto a secure server. This study was approved by Touro University Institutional Review Board (PH-0220). All methods were performed in accordance with the Declaration of Helsinki.

## Results

The study sample comprised 369 FEWs, of whom 16.8% reported losing all income, and 83.2% lost partial income due to the COVID-19 pandemic (Table 1). The mean age (31.6 vs. 30.6), years of formal education (6.3 vs. 6.3), marital status (24.2 vs. 23.8 never married), and the number of children (1.3 vs. 1.1) of women reporting having lost all income were not significantly different from those who experienced partial income loss. Participants who lost total income reported significantly lower daily access to the internet and telephone or tablet. The total income loss group also reported a significantly lower average number of dependent family members since 2020 and had lower average income. Food insecurity, mental ill health, and GBV experiences were all significantly higher among the total-income-loss group than those with partial income loss.

The results of our multivariable logistic regression analysis are displayed in Table 2. Controlling for the number of dependents, smartphone ownership, and food security, the odds ratio (OR) for GBV was significant in the adjusted model (AOR = 1.23 (1.08–1.40), *p* = 0.001), indicating that those who experienced total income lost were more likely to experience GBV than those who experienced partial income loss. In addition, they were significantly less likely to be food secure (*p* = 0.044), less likely to own a smartphone (0.023), and had more dependents (< 0.001).

**Table 2:** Multivariable logistic regression analysis of total income loss and GBV experiences

**Variable**	**AOR (95% CI)**	***P*-value**
GBV score	1.23 (1.08–1.40)	0.001
Food security score	0.90 (0.80–0.99)	0.044
Smartphone ownership	0.59 (0.47–0.74)	0.023
Number of dependents	1.45 (0.16–12.75)	< 0.001

*CI* confidence interval, *GBV* gender-based violence

**Fig. 1:**
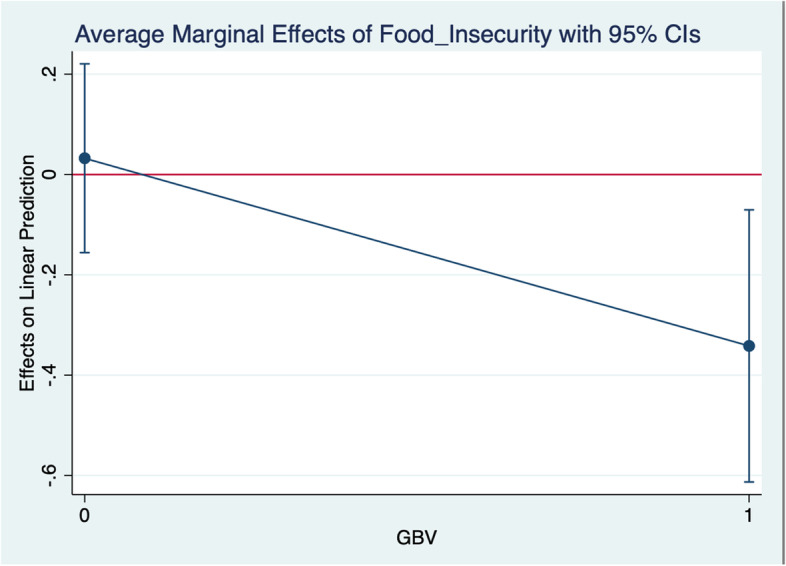
Average marginal effects of food security on gender-based violence

We found an interaction between GBV experiences and food security. Among the total-income-loss group, the more food-insecure FEWs experienced more GBV than the less food-insecure group (*p* = 0.007). We did not find a significant interactive relationship among the partial-income-loss group. We display this relationship using average marginal effects in Fig. 1.

## Discussion

To our knowledge, this is the first study examining the relationships between income loss due to the COVID-19 pandemic, GBV, food security, and mental health among FEWs in Cambodia. Our results show that FEWs who had lost all income due to job loss from the COVID-19 pandemic experienced significantly higher GBV and food insecurity than FEWs who lost partial income. The association between income loss and mental health remained statistically insignificant after adjusting for potential confounders. We also revealed significant interactions between food security and GBV among participants who lost total income due to the COVID-19 pandemic.

Our findings are in line with the findings from the Cambodia NGO Committee on the United Nations’ Convention on the Elimination of All Forms of Discrimination Against Women (CEDAW) Monitoring report released in early 2021. The report stated that women did experience a disproportionate about of loss of income because more women work in the industries most affected by the economic shutdown including hospitality, entertainment, and restaurant industry than men. They also stated that economic pressure can lead to abusive situations for women [26].

Several studies have found that economic shutdown measures taken in response to the pandemic intensified pre-existing inequalities between men and women and led to increased violence experienced by women and girls [27, 28]. Some suggested pathways posit that more women experienced employment loss than men, with this relationship being exacerbated in the poorest regions of the world [28]. Increased violence and the diminished ability to negotiate safe sex have been noted in other studies focused on sex and entertainment workers during the pandemic in the United States [29], Kenya [30, 31], and India [32].

Supporting research has also shown that income loss during the pandemic resulted in food insecurity for many women and especially women in entertainment and sex work. Studies found that income loss and poverty led to a high probability of suffering from food insecurity, particularly for female-headed households during the pandemic in Cambodia [33]. Being a venue-based sex worker during the pandemic was associated with increased food insecurity in Singapore [34]. A study on the impact of pandemic measures on food insecurity in Malawi found that vulnerable residents in the informal sector, such as sex workers, faced high food insecurity because of job loss [35].

In addition, we found an interaction between food insecurity and GBV by income status in this study. We do not know the direction of the association of the variables due to the cross-sectional nature of the study. However, existing research has shown an interaction relationship suggesting that, since GBV affects women and girls in their economically productive years, it can compromise their capacity to generate income and re-enforcing the poverty cycle that leads to food insecurity [36]. Other cross-sectional studies found that female sex workers in Maryland with severe food insecurity had greater odds of physical intimate partner violence and homelessness [37]. Among a similar group in Canada, food insecurity was associated with recent violence and unstable housing [38].

### Limitations

Due to the cross-sectional nature of this study, we could not determine the causal relationships between income loss and adverse outcomes. It is also essential to consider that FEWs may not accurately recall the frequency they experienced specific episodes of GBV, food security, or mental health. Another limitation is that all measures were self-reported, which may introduce several types of bias, including social desirability, as participants may have answered in a more favorable manner. For example, FEWs may non-differentially underreport the frequency and degree of GBV, food insecurity, and mental ill-health they experienced.

Despite these limitations, the findings from this study have important implications for interventions aimed at improving food security and GBV among FEWs in Cambodia who had lost their income due to the global crisis. Governments should consider providing emergency assistance and support to entertainment and sex workers currently ineligible for government assistance. Partnering with community organizations, they should consider providing essential supplies such as non-perishable foods. In addition, cash transfers to families whose income is dependent on entertainment work may be an important policy to consider during lockdowns, especially for those left out of the government’s one-time cash transfer.

## Conclusion

We found that FEWs who had lost all their income experienced significantly more GBV and food insecurity. Disaster response initiatives should consider the implications of completely closing venues that provide survival income and physical security for vulnerable people. Safety measures and government support for these formal and informal workers should be prioritized in terms of safety, food access, and mental health. Food assistance programs should be prioritized for this vulnerable group during crisis periods. In anticipation of future pandemics, this study serves to inform governments and community leaders of the needs of this marginalized community so that they can be integrated into a longer-term protection strategy. 

## Data Availability

Data and materials are available upon request. Please contact the corresponding author, Dr. Carinne Brody at cbrody@touro.edu.

## Abbreviations

AOR: Adjusted odds ratio
CI: Confidence interval
COVID-19: Coronavirus 19
DAG: Directed acyclic graph
FEW: Female entertainment workers
GBV: Gender-based violence
GHQ: General Health Questionnaire
SD: Standard deviation
USD: United States dollar
USDA: United States Department of Agriculture
